# Effects of rich cannabidiol oil on behavioral disturbances in patients with dementia: A placebo controlled randomized clinical trial

**DOI:** 10.3389/fmed.2022.951889

**Published:** 2022-09-06

**Authors:** Vered Hermush, Liora Ore, Noa Stern, Nisim Mizrahi, Malki Fried, Marina Krivoshey, Ella Staghon, Violeta E. Lederman, Lihi Bar-Lev Schleider

**Affiliations:** ^1^Geriatric Wing, Laniado Hospital, Netanya, Israel; ^2^Technion School of Medicine, Haifa, Israel; ^3^Department of Graduate Studies in Health Systems Management, The Max Stern Yezreel Valley College, Jezreel Valley, Israel; ^4^Research Department, Tikun-Olam Cannbit Pharmaceuticals, Tel Aviv, Israel; ^5^Clinical Research Center, Soroka University Medical Center and Faculty of Health Sciences, Ben-Gurion University of the Negev, Be’er Sheva, Israel

**Keywords:** medical cannabis, cannabidiol, dementia, behavioral disturbances, agitation, randomized controlled trial (RCT), neuropsychiatric symptoms

## Abstract

**Background:**

Almost 90% of patients with dementia suffer from some type of neurobehavioral symptom, and there are no approved medications to address these symptoms.

**Objective:**

To evaluate the safety and efficacy of the medical cannabis oil “Avidekel” for the reduction of behavioral disturbances among patients with dementia.

**Materials and methods:**

In this randomized, double-blind, single-cite, placebo-controlled trial conducted in Israel (ClinicalTrials.gov: NCT03328676), patients aged at least 60, with a diagnosis of major neurocognitive disorder and associated behavioral disturbances were randomized 2:1 to receive either “Avidekel,” a broad-spectrum cannabis oil (30% cannabidiol and 1% tetrahydrocannabinol: 295 mg and 12.5 mg per ml, respectively; *n* = 40) or a placebo oil (*n* = 20) three times a day for 16 weeks. The primary outcome was a decrease, as compared to baseline, of four or more points on the Cohen-Mansfield Agitation Inventory score by week 16.

**Results:**

From 60 randomized patients [mean age, 79.4 years; 36 women (60.0%)], 52 (86.7%) completed the trial (all eight patients who discontinued treatment were from the investigational group). There was a statistically significant difference in the proportion of subjects who had a Cohen-Mansfield Agitation Inventory score reduction of ≥ 4 points at week 16: 24/40 (60.0%) and 6/20 (30.0%) for investigational and control groups, respectively (χ^2^ = 4.80, *P* = 0.03). There was a statistically significant difference in the proportion of subjects who had a Cohen-Mansfield Agitation Inventory score reduction of ≥ 8 points at week 16: 20/40 (50%) and 3/20 (15%), respectively (χ^2^ = 6.42, *P* = 0.011). The ANOVA repeated measures analysis demonstrated significantly more improvement in the investigational group compared to the control group at weeks 14 and 16 (*F* = 3.18, *P* = 0.02). Treatment was mostly safe, with no significant differences in the occurrence of adverse events between the two groups.

**Conclusion:**

In this randomized controlled trial, ‘Avidekel’ oil significantly reduced agitation over placebo in patients suffering from behavioral disturbances related to dementia, with non-serious side-effects. Further research is required with a larger sample size.

## Introduction

Dementia, characterized by a progressive decline in cognitive and functional abilities and challenging behavioral symptoms (1, 2), is one of the major causes of disability and dependency among older adults (3). Neuropsychiatric symptoms (NPS) occur in up to 90% of patients with dementia (4–6), and are associated with a reduced quality of life (7, 8). Symptoms contributing to decreased quality of life include agitation, mood disorders, hallucinations and delusions (psychosis), and sleep disorders (7, 9, 10). Agitation, a common NPS in dementia, is associated with an increased rate of cognitive and functional decline (11), rapid disease progression (12, 13), and an earlier death (14) compared to patients with dementia without agitation. In addition, patients with agitation are more likely to be admitted to institutions (15–18), and to require more antipsychotics and antidepressants (19), increasing the overall cost of care. Meta-analyses on the reasons patients with dementia are placed in nursing homes confirm the significant role of NPS symptoms that are ineffectively managed (20, 21).

In the absence of approved medications for NPS, antipsychotics are typically used off-label to treat agitation in dementia, although evidence for their efficacy is limited and usage may involve dangerous side-effects (22–26). A recent meta-analysis found an increased odds of cerebrovascular events, fracture, and death associated with antipsychotics; increased odds of falls associated with dextromethorphan-quinidine; and increased odds of death associated with anticonvulsants (22). Guidelines recommend the use of antipsychotics for the treatment of NPS in patients with dementia only when symptoms are dangerous or cause significant patient distress (22). Identifying an effective, low-risk therapeutic alternative for NPS, and specifically agitation, in patients with dementia is essential.

Cannabinoids work by interacting with receptors in the endocannabinoid system (ECS), especially cannabinoid 1 receptor (CB1R) and cannabinoid 2 receptor (CB2R). CB1Rs are extensively distributed throughout the body, with a significant presence in the central nervous system, whereas CB2Rs are found in immune cells and tissues (27). The ECS is an important neuromodulatory system linked to a variety of psychiatric, neurodegenerative, and motor illnesses, including schizophrenia, anorexia, Alzheimer’s disease, Parkinson’s disease, and Huntington disease (28, 29). Delta-9-tetrahydrocannabinol (THC) and cannabidiol (CBD) are the two most common cannabinoids found in the cannabis plant (30). CBD has anti-inflammatory, neuroprotective, antipsychotic, anxiolytic, and antidepressant properties (31). While THC is the primary psychoactive ingredient (32), CBD is non-intoxicating (30); and when combined with THC, may counterbalance the psychoactivity of THC (33). While each of the two main cannabinoids has been linked to clinical and physiological effects on its own, researchers have hypothesized that the main cannabinoid and minor cannabinoids operate synergistically (34). Several controlled studies suggest that CBD is safe and effective for the treatment of anxiety (35–38), Parkinson’s disease (37, 39), post-traumatic stress disorder (38), autism (40), epilepsy (41), and schizophrenia (42). Some clinical data supports the beneficial therapeutic effects of cannabinoids on behavioral symptoms, particularly on agitation in patients with dementia (43–45); however, reviews concluded that it is uncertain whether cannabinoids have any beneficial or harmful effects on behavioral disturbances related to dementia. All included studies tested THC and synthetic THC analogs; none of them examined the effect of CBD on agitation (46, 47). Although treatment with cannabinoids appears to be safe in patients with dementia (47), cancer (48), and older patients (49); overall evidence for the management of dementia-related NPS with medical cannabis has been equivocal (50). As CBD cannabis oils are becoming increasingly available, the need for further evaluation of CBD cannabis oils as a possible treatment option for agitation and identification of the treatment characteristics is increasing.

The primary objective of this trial was to evaluate the safety and efficacy of cannabis oil extracted from one chemovar “Avidekel” (30% CBD and 1% THC: 295 mg and 12.5 mg per ml, respectively), for behavioral disturbances in patients with dementia.

## Materials and methods

### Study design

This was a single-center, randomized (2:1), placebo-controlled, double-blind trial. Patients were recruited nationally by the principal investigator (VH). During enrollment, written informed consent was provided by the legal representatives of all participants, and an application for a cannabis treatment license was arranged (issuance took an average of seven weeks). Over 16 weeks of the treatment period, participants came in for follow-up every two weeks, with the option to terminate their participation. After completing the study, all trial participants were offered the option to renew their cannabis treatment license. The trial took place in a tertiary hospital in Israel from December 2017 to September 2019.

The trial, registered in ClinicalTrials.gov: NCT03328676, was approved by the Laniado Hospital Ethics Committee (project LND 0111-16) and the clinical trials department at the Israel Ministry of Health (project 20173138). Study procedures were conducted in accordance with the Declaration of Helsinki and the International Conference on Harmonization Consolidated Guidelines on Good Clinical Practice and followed the CONSORT reporting guideline (51).

### Participants

During screening, participants were evaluated for eligibility criteria, which included an age of 60 years or older, diagnosis of a major neurocognitive disorder according to the DSM-5 criteria (all types of dementia), Mini–Mental State Examination (MMSE) (52) score of < 26 for cognitive impairment measurement, clinically relevant neuropsychiatric behaviors defined as Neuropsychiatric Inventory–Nursing Home Version (NPI-NH) (53–55) sub-score of agitation ≥ 3, a stable medication regimen for at least two weeks prior to baseline visit, and residence in either an institutionalized setting or in a non-institutionalized setting subject to 24-h supervision. Exclusion criteria included severe heart disease [New York Heart association (NYHA) class IV] (56), epilepsy, anxiety disorder; psychotic conditions in the present or in the past (not related to dementia), family history of schizophrenia, current substance use disorder, recent cannabis experience, or scheduled surgery during the trial.

### Randomization

Eligible participants were randomly assigned by a computerized random-number generator system in a 2:1 ratio to receive either ‘Avidekel’ oil or a placebo. Patients with dementia are required to have consent of legal representatives in order to enroll in clinical trials. To encourage caregivers’ interest in enrollment of this trial, the 2:1 ratio was employed (57). The randomized list of patients was set before the trial was initiated, and the investigational product (IP) and placebo were prepared. Patients, families, and the medical teams were masked to the individual patients’ treatment assignment. To ensure masking was maintained, “Avidekel” and placebo oils were manufactured to have an identical appearance and smell.

### Investigational product

The IP or placebo was added to the routine medication regimen (Table 1). Subjects received the IP or the placebo as drops applied under the tongue three times a day. Participants in the investigational group received “Avidekel” (made in Israel by Tikun-Olam Cannbit Pharmaceuticals), an ethanol extraction of rich CBD (∼15%), low-THC (∼0.5%) cannabis chemovar dissolved in olive oil. The IP contained 30% CBD, 1% THC, 1% Cannabichromene (CBC), 0.5% Cannabigerol (CBG), and 0.5% Cannabidivarin (CBDV). One drop of 0.04 ml contains 11.8 mg CBD and 0.5 mg THC. Patients in the control group received a placebo containing olive oil and chlorophyll.

**TABLE 1:** Characteristics of the patient population at baseline.

Characteristic	Avidekel oil (*n* = 40)	Placebo oil (*n* = 20)	P value
**Age (years), mean ± SD**	78.8 ± 9.3	80.5 ± 9.6	0.51
**Gender, n (%)**			
Females	22 (55)	14 (70)	0.26
Males	18 (45)	6 (30)	
**Country of birth, n (%)**			
Israel	12 (30)	6 (30)	0.95
Other	28 (70)	14 (70)	
**Residence, n (%)**			
Institution	7 (17)	2 (10)	0.66
Home	33 (83)	18 (90)	
**Years since diagnosis, mean ± SD**	4.24 ± 2.91	3.27 ± 2.42	0.27
**Comorbidities, n (%)**			
Cardiovascular diseases	33 (83)	17 (85)	0.81
Hypertension	17 (43)	9 (45)	0.85
Diabetes-type 2	11 (28)	6 (30)	0.84
Neurologic^1^	10 (25)	8 (40)	0.23
Endocrine^2^	5 (13)	2 (10)	0.78
Eye/ear	5 (13)	2 (10)	0.78
Depression	3 (8)	1 (5)	0.71
Renal	2 (5)	2 (10)	> 0.99
Other	16 (40)	9 (45)	0.71
**Medication, n (%)**			
Antihypertensive	21 (53)	12 (60)	0.85
Antidepressant	21 (53)	7 (35)	0.14
Antipsychotic	17 (43)	9 (45)	> 0.99
Sedative	12 (30)	10 (50)	0.46
Other	31 (78)	20 (100)	0.47
**Questionnaires, mean ± SD**			
MMSE^3^ score	12.4 ± 6.8	15.2 ± 6.2	0.13
CMAI^4^ score	59.3 ± 20.3	58.7 ± 22.3	0.92
NPI-NH^5^ score	41.7 ± 19.1	42.5 ± 20.1	0.88
GDS^6^ score	4.9 ± 3.3	2.8 ± 3.1	0.02
PAINAD^7^ score	0.1 ± 0.4	0.1 ± 0.4	0.99
CGI-S-A/A^8^ score	2.6 ± 3.3	2.9 ± 3.3	0.74

Data are presented as mean ± standard deviation for continuous data and No. (%) for categorical data. SD, Standard Deviation.

^1^Neurologic co-morbidities include cerebrovascular disease and epilepsy.

^2^Endocrine co-morbidities include hypothyroidism and hyperthyroidism.

^3^MMSE – Mini–Mental State Examination. Range and scaling: 0–30 points (≤ 9 meaning severe cognitive impairment).

^4^CMAI – Cohen-Mansfield Agitation Inventory. Range and scaling: 29–203 points (29 meaning no symptoms).

^5^NPI-NH – Neuropsychiatric Inventory–Nursing Home Version. Range and scaling: 0–144 points (0 meaning no symptoms).

^6^GDS – Geriatric Depression Scale. Range and scaling: 0–30 points (0 meaning no symptoms).

^7^PAINAD – Pain Assessment in Advanced Dementia Scale. Range and scaling: 0–10 points (0 meaning no symptoms).

^8^CGI-S-A/A – Clinical Global Impression for Agitation and Aggression. Range and scaling: 0–10 points (0 meaning no symptoms).

Caregivers were instructed to shake the oil bottle, place the drops of oil with a tablespoon under the patient’s tongue, and wait one minute before swallowing to enhance oil absorption. The initial dose was one oil drop in the morning, afternoon, and evening, for two days. They were instructed to increase each dose by one drop in increments of two days. The dose was titrated gradually depending on the tolerance of each patient, to a maximum dose of 21 drops per administration or until an adverse reaction occurred. The caregivers were instructed to then taper down one level to a pre-adverse reaction dose. The time for each patient to “find” the therapeutic dose: a balance between maximum reduction in agitation and minimum side-effects, lasted up to six weeks. After the titration phase, patients entered a ten-week treatment phase of fixed-dose (Supplementary Table 1).

We selected this specific chemovar “Avidekel” aiming to minimize side-effects. This was based on earlier clinical experience with 39 patients with indications for dementia and on 93 patients with pediatric autism spectrum disorder with behavioral disturbances (58). In both cases, patients receiving this product showed improvement in agitation with low-frequency side-effects. This type of sublingual administration (59) is more accurate with fewer fluctuations than other routes of administration. A similar product was tested for pharmacokinetics parameters in Crohn’s disease patients and demonstrated blood concentrations of the main active ingredients and their metabolites (60).

### Safety assessments

For safety evaluation, serious adverse events (SAEs; defined as: death, life-threatening events, hospitalization, debilitation, or immobility), and all adverse events (AEs), with a severity score on a Likert scale of 1 to 10, were collected in all trial visits. In this population with many comorbidities and medications, the symptom list of main AEs was also evaluated at baseline and documented as a non-IP-related AE report. An AE was defined as any unfavorable symptom, sign, syndrome, or disease that occurred during the study, having been absent at baseline, or, if present at baseline, appeared to worsen. Clinical data included vital signs and physical examination information collected in all trial visits, as well as blood chemistry and hematology labs collected every other visit.

### Outcomes measures

The primary efficacy endpoint was the proportion of subjects achieving a 4-point decrease in the Cohen-Mansfield Agitation Inventory (CMAI) at week 16 compared to baseline (61–65). A total CMAI score was obtained by summing all items from a caregivers’ rating questionnaire consisting of 29 agitated behaviors, each rated on a 7-point scale of frequency, with higher scores indicating greater severity. A total score of > 45 was regarded as clinically significant agitation, and a total reduction of 8 points or more was considered a clinically significant change (65). We determined that a 4-point decrease in CMAI score represents a better outcome compared to a similar randomized controlled trial that used oral THC (in which a 2.3 points reduction in the active group was not significant) (66), and above the placebo effect of two points decrease in the CMAI (67).

Secondary outcomes included: The proportion of subjects achieving an 8-point decrease in mean CMAI score, proportion of patients achieving 30% and 50% reduction in CMAI scores, the time necessary to achieve a 4-point reduction in CMAI, mean change in NPI-NH agitation/aggression sub-score. In the NPI-NH, the higher the score, the more severe and frequent the behavioral disturbances. The following questionnaires were also administered: the Geriatric Depression Scale (GDS), the Pain Assessment in Advanced Dementia Scale (PAINAD), the Clinical Global Impression for Agitation and Aggression (CGI-S-A/A), and the MMSE.

At each visit, a geriatrician and a trained occupational therapist examined the patients. All study questionnaires were administered and completed on paper by the trained staff and answered by the patient’s main caregiver (a family member or a hired caregiver) on every visit and recorded to an electronic Case Report Form.

### Statistical analysis

Sample size was calculated using the Power and Precision version 4 software (68), for a power of 80% and for two-sided α level of 0.05 to detect a difference in the proportion of successful reduction in CMAI scores between the investigational group compared to control at week 16. Success was defined as at least a 4-point reduction. For an expected difference of 35% in the proportion of success between the groups, an unbalanced sample of 42 and 22 was selected for the investigational and placebo group, respectively. Thus, 64 patients were randomly assigned to the investigational or control group (4 patients withdrew immediately after randomization, leaving 60 patients who started treatment to be included in the analysis). A 35% difference in proportion between the two groups was selected based on findings from an un-published report on the IP that was used to treat 14 patients with dementia-related behavioral disturbances.

The efficacy analyses were performed according to the intention-to-treat (ITT) principle, in order to provide unbiased comparisons among groups. The ITT analysis was done in all patients randomized and receiving treatment, with missing data imputation for patients who did not complete the trial (using last-observation-carried-forward method). We further performed a per protocol (PP) analysis (for 52 patients) as a sensitivity analysis, in which only patients who completed the trial according to protocol and had data available from all time points were counted toward the results. The primary outcome, CMAI reduction of >8 points and proportion of patients achieving 30% and 50% reduction in CMAI scores, were analyzed with the chi-square test including Yates’ corrected chi-square (continuity correction).

The baseline CMAI distribution was tested for normality using the Kolmogorov-Smirnoff test. The Mauchly’s Test of Sphericity was used to test whether the variances of the differences were equal. Baseline characteristics between groups are presented as means and standard deviations for continuous variables and as frequencies and percentages for categorical variables. Chi-square tests and independent *t*-tests were performed to compare groups for categorical and continuous baseline variables, respectively.

The GLM (general linear models) ANOVA Repeated Measures procedure was used to provide an analysis of variance for repeated CMAI measurements for nine visits on each subject. The analyses involve one within factor (time) and one between factor (groups). Changes over time and differences within groups were calculated (time*group), including contrasts tests to test differences among factor levels (1 factor, 9 levels), with a total significant level of 5%. Mauchly’s Test of Sphericity indicated that the assumption of sphericity had been violated [χ^2^(35) = 353.4, *P* < 0.001 for ITT and χ^2^(35) = 299.4, *P* < 0.001 for PP], meaning the F-statistic is positively biased rendering it invalid and increasing the risk of Type I error. To overcome this problem, we corrected the degrees of freedom using the Greenhouse-Geisser correction to obtain a valid critical F-value. The contrast was compared by method: difference, each level was compared to baseline. Analyses were performed on two full data sets (without missing data), the ITT set (*n* = 60) and the PP set (*n* = 52). In addition, the GLM test was performed again with a *post hoc* analysis based on the MMSE score to compare the change in CMAI in patients with higher or lower score than the median MMSE score.

Kaplan Meier survival analysis was performed to compute the time to achieve a CMAI ≥ 4-point reduction (success) for each group and the group difference was tested using the log rank chi-square test. Comparison of CMAI mean score between the two groups was analyzed by the independent *t*-test.

Comparison between groups in NPI-NH frequencies of all sub-categories (as dichotomous variables: yes/no) were analyzed by the Fisher’s exact test for baseline and end of study. NPI-NH factors scores, total NPI-NH, and all other variables were tested by independent *t*-test. Frequency of AEs and medications consumption between the two groups was compared by using the Fisher’s exact test.

Data were analyzed with IBM SPSS statistics software version 27.0 (SPSS Inc. Headquarters, Illinois, United States). Significance levels were set at 0.05.

## Results

**FIGURE 1:**
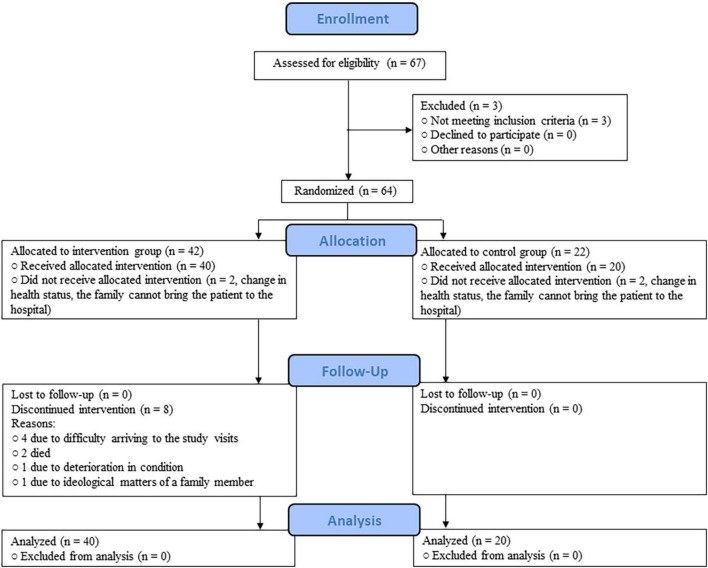
CONSORT diagram.

Of 67 patients screened for a possible enrollment, three patients were not eligible and four opted not to participate in the trial. Among the 60 randomized patients initiating treatment, the mean age was 79.4 ± 9.4 years; 36 (60.0%) were female and 52 (86.7%) completed 16 weeks of trial (Figure 1).

Upon enrollment, no meaningful differences were found. At baseline, all recruited patients presented MMSE scores of ≤ 25 (Table 1). In the repeated measures analysis, there was no difference in MMSE change from baseline to week 16 between the two groups (*F* = 1.58, *P* = 0.21). Overall, 32 of 40 participants in the investigational group (80.0%) and all participants in the control group completed the 16-week treatment. Two patients died of non-product-related causes. For the remaining six patients, attrition seemed due to personal and caregiver difficulties. AEs were not reported as a reason to leave the trial. At baseline, there were no statistically significant differences between completers and those who did not completed the trial.

Participants in the active and control groups consumed on average 14.9 and 17.9 drops per administration, respectively (44.7 and 53.7 drops per day, respectively) (Supplementary Figure 1). Mean CBD and THC consumption per administration was 175.8 mg and 7.4 mg, respectively (527.5 mg and 22.3 mg per day, respectively) (Supplementary Figure 2). Dose was not correlated with age (*r* = –0.17, *P* = 0.28) or with the outcome, both the change in CMAI (*r* = –0.23, *P* = 0.21), and the reductions of ≥ 4 point (*t* = 0.21, *P* = 0.83).

### Primary outcome

The primary endpoint of the trial was the proportion of subjects achieving a CMAI ≥ 4-point decrease during the treatment period. For the ITT set, the proportions observed were 24/40 (60.0%) and 6/20 (30.0%) for investigational and control groups, respectively (χ^2^ = 4.80, *P* = 0.03; with continuity correction χ^2^ = 3.67, *P* = 0.06). For the PP set (52 completers), the proportions observed were 22/32 (68.7%) and 6/20 (30.0%) for investigational and control groups, respectively (χ^2^ = 7.44, *P* = 0.006; with continuity correction χ^2^ = 5.96, *P* = 0.01).

### Secondary outcomes

**FIGURE 2:**
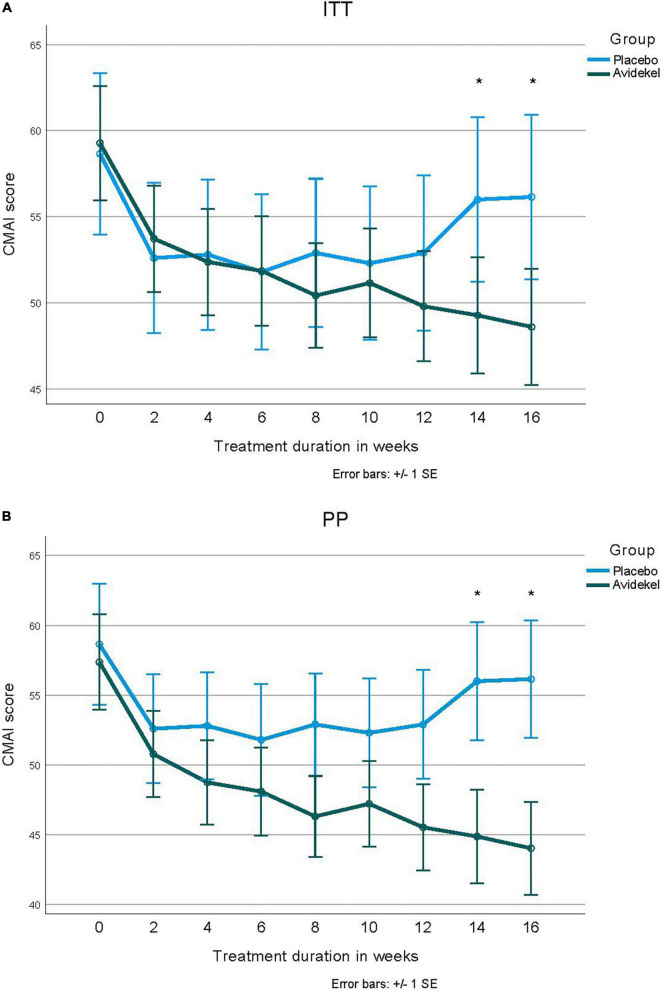
The Cohen-Mansfield Agitation Inventory score reduction over time between groups. Panels **(A,B)** present mean CMAI scores in the two groups, throughout the trial visits both in intention-to-treat analysis of all randomized patients that initiated treatment (*n* = 60), and per protocol analysis of patients who completed the trial according to protocol (*n* = 52). **(A)** Intention-to-treat analysis. **(B)** Per-protocol analysis.

The main hypothesis that the consumption of the IP will reduce behavioral disturbances and restlessness in older patients with dementia was tested by the CMAI reduction over time between groups (Figure 2). The CMAI baseline measures were slightly skewed, but we were unable to observe a significant skewed distribution when splitting into groups (Kolmogorov-Smirnoff *P* > 0.05). We compared the CMAI reduction from baseline to week 16 in both ITT and PP sets. Both demonstrate a significantly greater reduction in the investigational group, compared to the control group. In the ITT set, the reduction in CMAI scores at week 16 was of 10.7 ± 15.2 and 2.5 ± 9.4 points (*t* = –2.20, *P* = 0.03) for the investigational and control group, respectively. In the PP set, the reduction was of 13.3 ± 15.3 and 2.5 ± 9.4 (*t* = –2.85, *P* = 0.006) for the investigational and control group, respectively. The average CMAI score in the last visit for the investigational group was 44.03 ± 13.21. The CMAI aggressive behavior sub-score also showed significantly greater improvement in the investigational group compared to the control group (*t* = 1.30, *P* = 0.02 for the PP set). There was a statistically significant difference in the proportion of subjects who had a CMAI score reduction of ≥ 8 points at week 16: 20/40 (50%) and 3/20 (15%), respectively (χ^2^ = 6.425, *P* = 0.011). To test the reduction of CMAI over time, we used two full data sets: an ITT set and a PP set of completers. The GLM ANOVA repeated measures over time of CMAI scores for the ITT data demonstrate a significant decrease over time in the multivariate test for both groups (within-subject effect *F* = 4.74, *P* = 0.001). Analysis demonstrated a significantly greater improvement in the investigational group compared to the control group, for tests of week 14 (*F* = 6.13, *P* = 0.01) and week 16 (*F* = 7.07, *P* = 0.01) compared to baseline. The same analysis for the PP data demonstrates a significant decrease over time (F = 6.45, P < 0.001) and with different reduction trends between the two groups (*F* = 3.18, *P* = 0.02). Results present a wide confidence interval; however, tests of difference between groups at week 14 (*F* = 4.83, *P* = 0.03) and at week 16 (*F* = 4.84, *P* = 0.03) were significantly different.

We tested whether patients with lower MMSE scores (14 and below) were different from patients with MMSE scores of 15 or higher in the CMAI change through time. We could not find a significant difference in either the entire group (*F* = 1.73, *P* = 0.12) or within the interventional group alone (*F* = 1.23, *P* = 0.33). For patients in the investigational group who achieved a ≥ 4-point decrease in CMAI (60.0%), it took a mean of 8.8 weeks (95% CI: 6.7–11.1 weeks), whereas patients who received the placebo and achieved a ≥ 4-point decrease in CMAI (30.0%) took 12.9 weeks (95% CI: 10.2–15.6). This difference was significant (log rank χ^2^ = 5.19, *P* = 0.02).

Table 2 demonstrates the differences between groups in clinical parameter scores for completers at baseline and at the end of the trial. NPI-NH results demonstrate a significant reduction (29.4%) in agitation/aggression (χ^2^ = 5.98, *P* = 0.01) and a significant reduction (22.5%) in sleep disturbances (χ^2^ = 5.19, *P* = 0.03) in the investigational group compared to the control group, as well as a significant difference in the mean NPI-NH Agitation/Aggression factor score at week 16 (*t* = 2.01, *P* = 0.03). Chang was from 12.1 ± 5.8 to 6.7 ± 6.9 in the investigational group, and from 15.5 ± 7.8 to 11.4 ± 8.7 in the control group. There was no statistically significant difference in the GDS, PAINAD, CGI-S-A/A or MMSE questionnaires. An improvement of < 30% in CMAI total score was achieved by 24.3% of patients in the investigational group, and 10% in the control group (χ^2^ = 0.94, *P* = 0.30). Similarly, an improvement of < 50% in CMAI total score was achieved by 8% of patients in the investigational group and 0% in the control group (χ^2^ = 0.24, P = 0.54). The most improved behaviors of the CMAI questionnaire that improved in all patients in the investigational group included making physical sexual advances, throwing things, spitting, hurting themselves or others, tearing things or destroying property, intentional falling, eating/drinking inappropriate substances, and making verbal sexual advances.

**TABLE 2:** Effects on neuropsychiatric signs and symptoms for completers, at baseline and end of trial.

Variable	Baseline – week 0			End of trial – week 16		
	Avidekel	Placebo	P	Avidekel	Placebo	P
	(*n* = 32)	(*n* = 20)		(*n* = 32)	(*n* = 20)	
**CMAI^1^ Sub-cores, mean ± SD**						
Aggressive behavior	14.0 ± 4.4	14.7 ± 5.0	0.60	12.0 ± 3.2	16.0 ± 8.6	0.02
Physically non-aggressive behavior	23.7 ± 9.1	22.9 ± 9.1	0.76	17.3 ± 7.4	21.0 ± 9.7	0.13
Verbally agitated behavior	19.7 ± 9.9	21.1 ± 10.4	0.63	14.7 ± 6.8	19.2 ± 10.6	0.07
**CMAI total score, mean ± SD**	57.4 ± 17.4	58.7 ± 22.3	0.81	44.0 ± 13.2	56.2 ± 25.5	0.03
**NPI-NH**^2^ **sub-categories, n (%)**						
Delusion	16 (50)	9 (45)	0.78	6 (19)	6 (30)	0.35
Hallucinations	12 (38)	6 (32)	0.77	6 (19)	5 (25)	0.59
Agitation/Aggression	32 (100)	20 (100)	–	21 (66)	19 (95)	0.01
Depression/Dysphoria	24 (75)	15 (75)	0.26	18 (56)	9 (45)	0.43
Anxiety	21 (66)	14 (70)	0.89	14 (44)	11 (55)	0.43
Elation/Euphoria	2 (6)	7 (35)	0.02	2 (6)	2 (11)	0.61
Apathy/Indifference	25 (78)	14 (70)	0.53	21 (66)	14 (74)	0.55
Disinhibition	11 (34)	12 (60)	0.09	9 (28)	11 (55)	0.08
Irritability/Lability	25 (78)	15 (75)	0.87	21 (66)	13 (65)	0.96
Aberrant Motor Behavior	19 (59)	11 (55)	0.78	19 (59)	8 (40)	0.17
Sleep disturbances	21 (66)	15 (75)	0.55	9 (28)	12 (60)	0.03
Appetite and eating disturbances	18 (56)	10 (50)	0.78	9 (28)	8 (40)	0.37
**NPI-NH Factors Scores, mean ± SD**						
Agitation/Aggression	12.1 ± 5.8	15.5 ± 7.8	0.08	6.7 ± 6.9	11.4 ± 8.7	0.03
Depression	7.1 ± 4.8	8.0 ± 6.2	0.56	4.1 ± 4.7	5.8 ± 6.1	0.26
Psychosis	5.4 ± 7.3	3.4 ± 4.3	0.27	1.3 ± 2.3	2.7 ± 4.5	0.14
Psychomotor agitation	7.9 ± 6.3	9.1 ± 6.6	0.51	3.6 ± 4.1	6.5 ± 6.7	0.06
Apathy	8.7 ± 5.8	6.7 ± 5.1	0.21	5.8 ± 5.5	4.9 ± 3.8	0.52
**NPI-NH Total score, mean ± SD**	41.2 ± 18.4	42.5 ± 20.1	0.81	21.4 ± 16.9	31.2 ± 22.0	0.08
**MMSE**^3^ **Total score, mean ± SD**	12.2 ± 6.3	15.2 ± 6.2	0.10	10.4 ± 6.8	13.7 ± 7.5	0.21
**GDS**^4^ **Total score, mean ± SD**	5.5 ± 3.3	2.8 ± 3.1	0.01	4.9 ± 4.0	3.3 ± 4.4	0.18
**PAINAD**^5^ **Total score, mean ± SD**	0.1 ± 0.4	0.1 ± 0.4	–	0.0 ± 0.0	0.1 ± 0.2	–
**CGI-S-A/A**^6|^ **Total Score, mean ± SD**	2.8 ± 3.5	2.9 ± 3.3	0.84	1.3 ± 2.4	2.0 ± 2.9	0.35

SD, Standard Deviation.

^1^CMAI – Cohen-Mansfield Agitation Inventory. Range and scaling: 29–203 points (29 meaning no symptoms). Analysis was performed on all patients.

^2^NPI-NH – Neuropsychiatric Inventory–Nursing Home Version. Range and scaling: 0–144 points (0 meaning no symptoms). Analysis was performed on all patients.

^3^MMSE – Mini–Mental State Examination. Range and scaling: 0–30 points (≤ 9 meaning severe cognitive impairment). Analysis was performed on 46 patients, 29 patients in the Avidekel group and 17 in the control group.

^4^GDS – Geriatric Depression Scale. Range and scaling: 0–30 points (0 meaning no symptoms). Analysis was performed on 42 patients, 26 patients in the Avidekel group and 16 in the control group.

^5^PAINAD – Pain Assessment in Advanced Dementia Scale. Range and scaling: 0–10 points (0 meaning no symptoms). Analysis was performed on 46 patients, 29 patients in the Avidekel group and 17 in the control group.

^6^CGI-S-A/A – Clinical Global Impression for Agitation and Aggression. Range and scaling: 0–10 points (0 meaning no symptoms). Analysis was performed on all patients.

There was no statistically significant difference in medications used between groups and over time, demonstrating stable medication consumption throughout the trial in both groups (Supplementary Table 2).

### Adverse events

All withdrawals occurred in the investigational group. The reported reasons for withdrawals were: four patients discontinued treatment due to difficulty commuting to the study appointments (one patient completed baseline visit, two patients completed 2 weeks, and one completed 4 weeks); one patient left after 4 weeks due to the ideological concerns of her son; one patient withdrew after the baseline visit due to a deterioration in his condition (dialysis patient).

Thirteen SAEs included two deaths and eleven hospitalizations (Table 3). There were no significant differences in the occurrence of SAEs (9 and 4 in the investigational and control groups, respectively). The two deaths were in the investigational group. The first patient, 94 years old, suffering from colonic cancer and chronic renal failure, died from septic shock after completion of 4 weeks in the study. The second patient, 87 years old, experienced recurrent hospitalizations due to severe hyponatremia and anemia, for which he was recurrently intubated, and died from breathing difficulties (only baseline results were recorded). There was no statistically significant difference in the death rate between the two groups (active group 6.25% versus placebo group 0.0%, χ^2^ = 1.28, *P* = 0.52). We did not see a direct link between the SAEs and the IP.

**TABLE 3:** Patients experiencing adverse events^1^.

Variable	Avidekel (*n* = 37)	Placebo (*n* = 20)	P
**Serious adverse events, n (%)**			
Hospitalization	7 (19)	4 (20)	0.74
Death	2 (5)	0 (0)	–
**Adverse events, n (%)**			
Decreased memory	12 (32)	2 (10)	0.06
Hallucinations	8 (22)	1 (5)	0.08
Sleepiness	18 (49)	6 (30)	0.17
Dry mouth	5 (14)	5 (25)	0.17
Confusion and disorientation	17 (46)	6 (30)	0.18
Fear	9 (24)	3 (15)	0.34
Restlessness	10 (27)	7 (35)	0.39
Blurred vision	4 (11)	1 (5)	0.41
Dizziness	6 (16)	2 (10)	0.47
Weakness	8 (22)	5 (25)	0.54
Red/Irritated eyes	2 (5)	1 (5)	0.61
Increased heart rate	3 (8)	1 (5)	0.65
Psychoactive effects	3 (8)	1 (5)	0.65
Headaches	3 (8)	2 (10)	0.66
Slurred speech	6 (16)	3 (15)	0.99
Decreased concentration	0 (0)	1 (5)	**–**
Other	21 (57)	9 (45)	0.29
Patients experiencing any adverse event	34 (91)	18 (90)	0.99

^1^The analysis was performed on an intention-to-treat population. In three patients, there were no visits after treatment initiation and side-effect reports were not available.

Sleepiness (48.6%), confusion and disorientation (45.9%), and decreased memory (32.4%) were the most frequent complaints among participants in the investigational group. No significant differences were observed in the occurrence of AEs between groups (Table 3). However, in the investigational group there were notably higher rates of decreased memory (χ^2^ = 3.52, *P* = 0.06), hallucinations (χ^2^ = 2.72, *P* = 0.08), sleepiness (χ^2^ = 1.85, *P* = 0.17), and confusion and disorientation (χ^2^ = 1.42, *P* = 0.18). No change in pulse or blood pressure were observed throughout the study.

## Discussion

In this randomized placebo-controlled trial, we aimed to test the hypothesis that broad-spectrum rich CBD medical cannabis oil differs from a placebo in alleviating behavioral disturbances in patients with dementia. Patients in the investigational group experienced a significantly greater reduction in sleep disturbances, and in agitation and aggression sub-score using two different measurement tools. The improvements were accompanied with non-serious side-effects.

Agitation CMAI scores decreased significantly in the investigational group over the course of treatment. Over the years, CBD has been suggested to have a positive clinical effect in patients suffering from neurological conditions. It has been found to be effective in reducing anxiety (69), Parkinson’s disease related symptoms (39), disruptive behavior, and other autism related symptoms (40). CBD has been also found to be effective as an anticonvulsant (41). There are no studies describing the effect of CBD on behavioral disturbances in dementia patients. Existing studies only tested THC and its analogs. In our study, 31% of patients in the investigational group reached the maximum dose allowed of 10.5 mg THC and another 15.6% reached 10 mg THC per administration; therefore, a direct effect of THC contributing to the decrease of behavioral disturbances cannot be ruled out. On the other hand, controlled studies with THC administration as a single active compound for the management of behavioral disturbances in dementia patients showed no significant decrease (66, 70, 71). Some pre-clinical studies demonstrated that the compounds in Avidekel work synergistically and that the combination of active ingredients in the IP is responsible for the observed effect. If this is the case, administering one component as an isolated material would not reproduce the same effect (72, 73). The difference in the average CMAI scores between groups only became significant at week 14, highlighting the importance of patience in the first few months of treatment based mainly on CBD.

In one study on the effects of antipsychotic drugs (74) on behavioral disturbances in dementia, the primary end point proportions in the antipsychotic group compared to the control resemble the numbers we received. In some other antipsychotics studies using the CMAI tool (75–77), results were different, and they did not find significant improvement in agitation compared to placebo. There was an improvement in behavioral symptoms in 30% of patients in the control group. This improvement may be explained by the placebo effect and by the non-specific benefit of being enrolled in a trial (78), with thorough bi-weekly medical monitoring. In the NPI-NH, the results of our study demonstrate a significant reduction in agitation/aggression total scores and are close to the NPI-NH total scores obtained in the investigational and control groups from other antipsychotic drugs studies (74). Further investigation is required to explore rich CBD cannabis oil as a treatment option for agitation in patients with dementia, especially because the average CMAI score in the Avidekel group at 16 weeks was below the definition of clinically significant agitation.

Although the etiology of dementia-related agitation involves psychological and social components, it is often predominantly characterized by anatomical and neurochemical changes in the brain (79). In a review on the pharmacological treatment addressing the etiology of dementia-related agitation and aggression, pharmacological modulation of specific molecular targets was suggested as management options (80). Some of the proposed molecular targets are affected by CBD, which acts on more than 65 targets [for a review, see (81–83)]. The mechanisms of action underlying the direct and indirect effects of CBD on agitation involve the regulation of the serotonin 1A receptor, CB1Rs, the hypothalamic-pituitary-adrenal axis, anandamide, CB2Rs, and GABAA receptors (81, 82, 84). Animal models showed that chronic administration of CBD led to a reduction in inflammation and increased clearance of amyloid beta (85), while also reducing anxiety, depression, and stress-related behaviors (86).

Sleep disturbances in the NPI-NH were significantly lower in the investigational group at week 16 compared to baseline. This finding is in line with the published literature demonstrating the positive effect of THC on sleep, in the context of different medical indications, both in controlled (87–89) and uncontrolled studies (45, 90–92). Similar results were found in controlled studies on a combination of THC and CBD (93–95). As for the effect of CBD on sleep, one study showed that CBD does not impair sleep (96), and several uncontrolled studies have shown that CBD improves sleep (69, 97). In this study, 49% of the investigational group reported drowsiness as a side-effect. The improvement in behavioral disturbances along with the reduction in sleep disturbances raises concerns regarding the anesthetizing characteristics of the IP. However, IP consumption does not appear to be related to increased apathy, as there were no differences between groups in NPI-NH apathy scores.

The treatment appears to be relatively safe. Common adverse events included sleepiness, confusion and disorientation, restlessness, fear, weakness, and hallucinations, among others. The safety profile of CBD cannabis oil appeared to be high in other studies as well (98, 99), including in pediatric populations (100–102). Although not statistically significant, the higher rates of decreased memory, sleepiness, and hallucinations in the investigational group should be further explored. It may indicate that the dose of 10 mg THC per administration for patients with dementia may be too high, even when combined with an increased presence of CBD. The occurrence of reported AEs in patients who discontinued treatment were not different from the rest of the cohort. Although we did not find a link between the IP and the study discontinuation, we cannot exclude the possibility that the IP might have a tolerability barrier.

### Limitations

Our trial has several limitations. All eight patients who discontinued the treatment belonged to the investigational group and the sample size of 60 participants for our main outcome in an ITT analysis has a power of only 60%. The small number of participants, recruited in a single medical center, with no comparison between sub-types of dementia (Alzheimer’s disease, Lewy body and vascular dementia), made the study group highly heterogenous, providing limited ability to define the safety profile of the IP. However, heterogeneity, specifically in dementia patients, increases the importance of the results. Outcome measures did not include measures that would rule out functional impairment following treatment with a product containing THC, and pharmacokinetic indices of the IP were not collected in this trial. Although the GDS questionnaire has been shown to retain acceptable qualities when applied to older patients with dementia, it is a less sensitive questionnaire compared to the Cornell Scale for Depression in Dementia (103). Due to the limited availability of “Avidekel” in most countries, there is a lack in necessary research required to compare chemovars and to identify which specific compounds in “Avidekel” resulted in the superior effect “Avidekel” has shown over other chemovars in the clinic, with similar concentrations of THC and CBD. Subsequent research should also aim to identify new efficacious chemovars.

## Conclusion

Our findings suggest that rich-CBD cannabis oil may alleviate agitation in older patients with dementia. One trial is not enough to make conclusions on the safety and efficacy of broad-spectrum CBD. We recommend conducting a large scale randomized controlled trial on behavioral disturbances related to dementia and to compare clinical sub-types of dementia.

## Data availability statement

The raw data supporting the conclusions of this article will be made available by the principal investigator, without undue reservation.

## Ethics statement

The studies involving human participants were reviewed and approved by the Laniado Hospital Ethics Committee (project LND 0111-16) and the Clinical Trials Department at the Israel Ministry of Health (project 20173138). The patients/participants provided their written informed consent to participate in this study.

## Author contributions

VH and LB-L conceived the study, wrote the protocol, and drafted the manuscript. VH was the guarantor. All authors acquired, analyzed, or interpreted the data, and critically revised the manuscript for important intellectual content.
