# Supplementary material for: Effects of rich cannabidiol oil on behavioral disturbances in patients with dementia: A placebo controlled randomized clinical trial
Source: Front Med (Lausanne). 2022 Sep 6;9:951889. doi: 10.3389/fmed.2022.951889 (PMC9486160; doi:10.3389/fmed.2022.951889)
Supplement: Supplementary file 1 [file Data_Sheet_1.pdf]

Supplementary Material

1 Supplementary Figures and Tables

1.1 Supplementary Figures

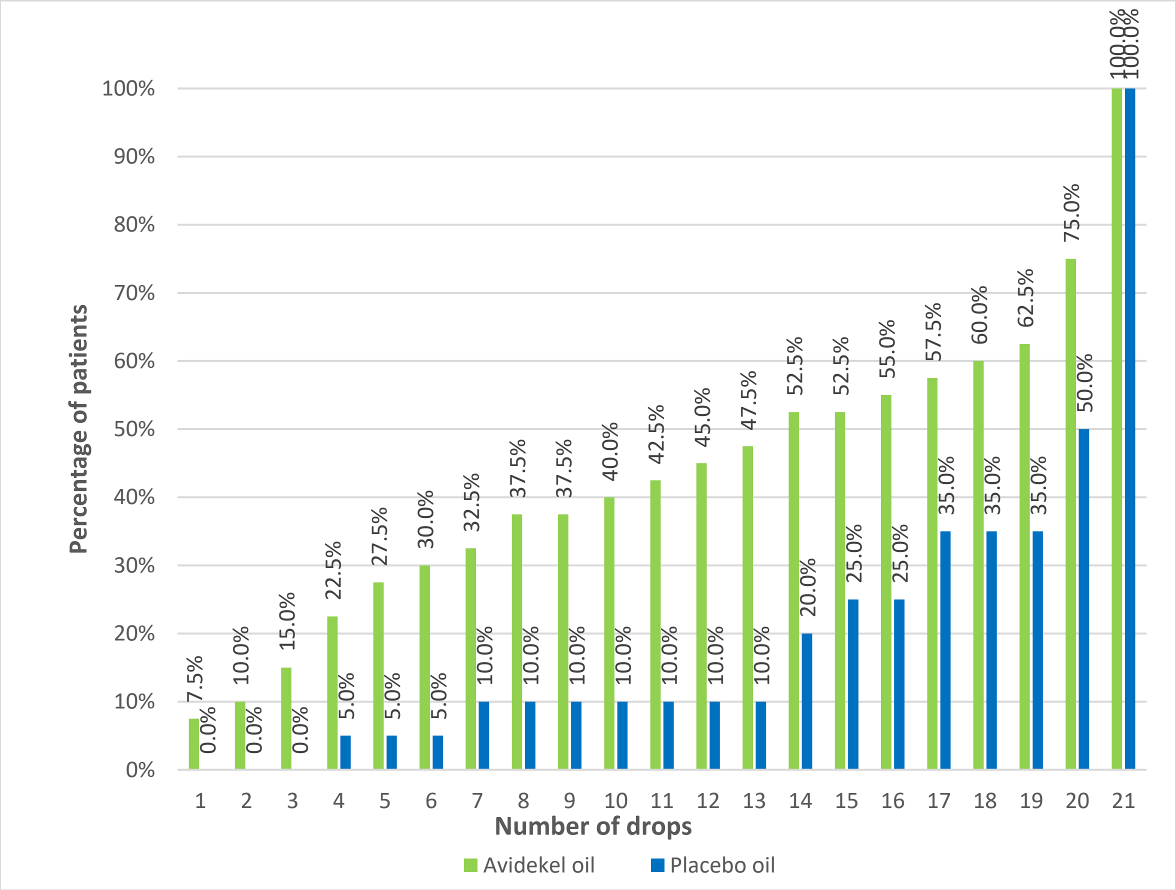

Supplementary Figure 1. Cumulative frequency for Avidekel oil and placebo oil consumption doses. The number of drops consumed after titration by 52 patients who completed the trial, receiving Avidekel oil or placebo oil at a single administration (there were three daily sublingual administrations). The investigational group and the placebo group consumed in average 14.9 and 17.9 drops per administration, respectively (44.7 and 53.7 drops per day, respectively). During titration in both groups, there were 13 cases of dose reduction and 10 cases of dose increase not by the protocol. After titration in both groups, there were two cases of dose reduction, one case of dose increase. There were 35 cases of skipped doses during the study.

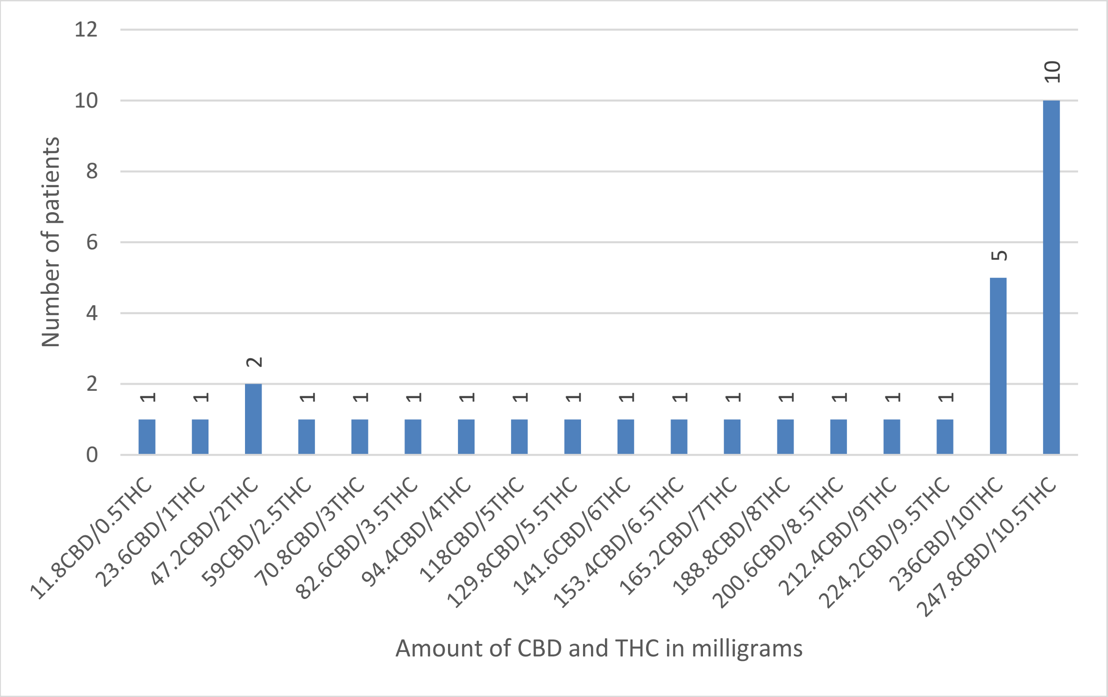

Supplementary Figure 2. CBD and THC Consumption. The CBD and THC consumption after titration of Avidekel oil in mg by 32 patients who ended the trial at single administration (there were three equal daily administrations). Mean CBD and THC consumption per administration was 175.8 and 7.4 mg, respectively (527.5 mg and 22.3 mg per day, respectively). One drop is equivalent to approximately 0.04 ml and contains approximately 11.8 mg CBD and 0.5 mg THC. The number of bottles each patient received at each visit was documented every visit by the research team. At the titration stage patients increased the dose according to the guidelines and after reaching the therapeutic dose, very few doses were skipped or deviated from the therapeutic dose, that remained relatively stable.

1.2 Supplementary Tables

Visits	1	2	3	4	5	6	7	8	9	10
**Time Point**	**Enrolment**	**Day 0 Baseline**	**Week 2 ± 3 days**	**Week 4 ± 3 days**	**Week 6 ± 3 days**	**Week 8 ± 3 days**	**Week 10 ± 3 days**	**Week 12 ± 3 days**	**Week 14 ± 3 days**	**Week 16 ± 4 days**
Informed Consent	X									
Inclusion/	X									
Demographics	X									
Medical History + Dementia history	X									
Physical Examination	X	X	X	X	X	X	X	X	X	X
Vital Signs*	X	X	X	X	X	X	X	X	X	X
**Assessments**										
Cohen-Mansfield Agitation Inventory (CMAI)	X	X	X	X	X	X	X	X	X	X
Neuropsychiatric Inventory (NPI-NH)	X	X	X	X	X	X	X	X	X	X

Clinical Global Impartation Severity-Agitation / Aggression (GAI-S-A/A)	X	X	X	X	X	X	X	X	X	X
Geriatric Depression Scale (GDS)	X	X			X			X		X
Pain Assessment in Advanced Dementia (PAINAD)		X	X	X	X	X	X	X	X	X
Mini-Mental State Examination (MMSE)		X			X			X		X
Adverse Events	X	X	X	X	X	X	X	X	X	X
**Blood Tests**										
Hematology panel	X		X		X		X		X	
Chemistry panel	X		X		X		X		X	
**IP Administration**										
Randomization	X									
IP Accountability			X	X	X	X	X	X	X	X
Concomitant	X	X	X	X	X	X	X	X	X	X
Titration	X	X	X	X	X					
Fix dose						X	X	X	X	X

*Vital signs - the following parameters will be collected: temperature, pulse, blood pressure, height (only screening visit) and weight.

Supplementary Table 3. Schedule of events.

Timeline	No. %									
	Antihypertensive		Antidepressant		Antipsychotic		Relaxing		Other	
	Avidekel	Placebo	Avidekel	Placebo	Avidekel	Placebo	Avidekel	Placebo	Avidekel	Placebo
Baseline	21 (65.6)	12 (60.0)	21 (65.6)	7 (35.0)	17 (53.1)	9 (45.0)	12 (37.5)	10 (50.0)	31 (96.9)	20 (100)
Week 2	21 (65.6)	12 (60.0)	18 (56.3)	8 (40.0)	16 (50.0)	9 (45.0)	12 (37.5)	10 (50.0)	31 (96.9)	20 (100)
Week 4	21 (65.6)	14 (70.0)	18 (56.3)	7 (35.0)	15 (46.9)	9 (45.0)	12 (37.5)	9 (45.0)	30 (93.8)	20 (100)
Week 6	18 (56.3)	14 (70.0)	17 (53.1)	7 (35.0)	14 (43.8)	8 (40.0)	13 (40.6)	9 (45.0)	30 (93.8)	20 (100)
Week 8	18 (56.3)	14 (70.0)	21 (65.6)	7 (35.0)	16 (50.0)	8 (40.0)	13 (40.6)	8 (40.0)	31 (96.9)	20 (100)
Week 10	18 (56.3)	14 (70.0)	19 (59.4)	4 (20.0)	15 (46.9)	9 (45.0)	13 (40.6)	10 (50.0)	31 (96.9)	20 (100)
Week 12	19 (59.4)	13 (65.0)	18 (56.3)	4 (20.0)	19 (59.4)	10 (50.0)	14 (43.8)	10 (50.0)	31 (96.9)	20 (100)
Week 14	19 (59.4)	13 (65.0)	19 (59.4)	4 (20.0)	19 (59.4)	11 (55.0)	15 (46.9)	11 (55.0)	31 (96.9)	20 (100)
Week 16	19 (59.4)	12 (60.0)	16 (50.0)	4 (20.0)	19 (59.4)	11 (55.0)	14 (43.8)	11 (55.0)	31 (96.9)	20 (100)

Supplementary Table 2. Concomitant medications over time for patients who completed the trial per protocol (Avidekel, N=32; Placebo, N=20).
